# Study of a Conical Plasma Jet with a Cloth-Covered Nozzle for Polymer Treatment

**DOI:** 10.3390/polym15163344

**Published:** 2023-08-09

**Authors:** Felipe Vicente de Paula Kodaira, Ana Carla de Paula Leite Almeida, Thayna Fernandes Tavares, Antje Quade, Luis Rogério de Oliveira Hein, Konstantin Georgiev Kostov

**Affiliations:** 1 School of Engineering, São Paulo State University (UNESP), Guaratinguetá 12516-410, SP, Brazil; 2 Leibniz Institute for Plasma Science and Technology—INP, 17489 Greifswald, Germany; quade@inp-greifswald.de

**Keywords:** APPJ, polymer treatment, conical APPJ, surface modification, atmospheric plasma

## Abstract

Although atmospheric pressure plasma jets (APPJs) have been widely employed for materials modification, they have some drawbacks, such as the small treatment area (couple of cm^2^). To overcome this limitation, a funnel-like APPJ with a wide exit has been proposed. In this work, a gas-permeable cotton cloth covered the nozzle of the device to improve the gas flow dynamics and increase its range of operation. The funnel jet was flushed with Ar, and the plasma was ignited in a wide range of gas flow rates and the gap distances between the exit nozzle and the sample holder. The device characterization included electric measurements and optical emission spectroscopy (OES). To evaluate the size of the treatment and the degree of surface modification, large samples of high-density polyethylene (PE) were exposed to plasma for 5 min. Afterward, the samples were analyzed via water contact angle WCA measurements, scanning electron microscopy (SEM), and X-ray photoelectron spectroscopy (XPS). It was found that surface modification occurs simultaneously on the top and bottom faces of the samples. However, the treatment incorporated different functional groups on each side.

## 1. Introduction

Atmospheric pressure plasma jets (APPJs) emerged by the end of the 1990s as an alternative to the conventional dielectric barrier discharge (DBD) systems that are prone to processing thin films and flat substrates, but are inadequate for treating 3D structures. APPJs are low-cost devices where electric discharge is ignited in a noble gas that flows through a dielectric capillary and the resulting plasma is ejected into the surrounding environment (usually ambient air) [1]. There, the energetic plasma species (electrons, photons, and metastables) interact with air molecules, creating reactive oxygen and nitrogen species (RONS) that are driven by the gas flow to a target where surface modification/decontamination can take place [2]. Normally, APPJs generate several cm long plasma plumes that can be easily adapted to treat irregular 3D objects and internal surfaces of narrow tubes or cavities [3,4]. In particular, the RONS generated by atmospheric plasma jets have shown to be very effective in improving the wettability of polymers [5,6]. Plasma jets are very versatile devices that can be driven by pulsed DC, AC, RF, and microwave sources, with diverse designs and operation parameters [1]. Moreover, the gas temperature of the plasma plume can be kept quite low (under 40 °C in some cases), which makes the plasma jets suitable for medical applications and the treatment of thermo-sensitive materials [4]. In summary, the simple construction, flexible geometry, and low cost make APPJ very attractive for use in diverse fields, ranging from material synthesis, surface modification, gas conversion, to treatment of liquids through biomedical and agricultural applications [1,2]. However, the plasma plume diameter is determined by the inner diameter of the capillary [7], (normally in the order of a few mm), which combined with the limited lifetime of reactive species results in a small area (typically several cm^2^) that can be covered by RONS [8], and eventually modified. Moreover, within the plasma-treated area (usually with a circular shape), the degree of material surface modification is not uniform, being more intense at the center (where the plasma jet hits the target) and less pronounced at the edges [9]. These issues may not be a problem in the case of a localized treatment, like plasma medicine, but they are serious drawbacks in the field of material processing. To remedy these disadvantages, different approaches, like target/jet manipulation [10], employing arrays of many plasma jets [11], different electrode configurations [12,13,14,15], high discharge frequency [16,17], shielding gases [9,18], the cooperative merging of several small plasma jets [19,20,21], and the use of a larger dielectric tubes [22] with different geometries [23,24] have been proposed. These methods help extend the plasma modification effect over a larger area (up to tens of cm^2^), but all of them have drawbacks. For instance, one common disadvantage of the large tube diameter [22], the cooperative jets merging [19,20], and the plasma jet arrays [11] is that those devices usually rely upon excessively high flow rates (~10 L/min) of helium gas. Moreover, due to the complex gas flow dynamics in irregular tubes [24] and the complex interaction between individual jets in a plasma array [25], ensuring uniform surface modification is very hard. In some excitation schemes like in [5,12,15,16], a long (up to 10 cm), brush-type plasma jet was generated, which, combined with a one-dimensional target movement, can provide uniform treatment over a large rectangular area [16]. Nevertheless, as shown in [12,16], the discharge is prone to instabilities that perturb plasma plume distribution along the gas duct, thus compromising the treatment uniformity. In [10], the authors report on an especially designed mechanical system for simultaneous jet rotation and tilting, which can provide uniform decontamination over a circular area (~20 cm^2^). Overall, the jet/target manipulation systems help achieve a larger treatment area, but they also add more process parameters and increment the device cost. To increase the size of plasma-modified surfaces, some authors proposed different shielding gases [9] and lateral He gas streams [18]. Once again, these new components increase the process cost and system complexity. In previous work, we proposed a simple plasma jet system terminating with a conical horn exit, which can perform uniform surface modification over the entire area covered by the horn [26], without any target/jet manipulation. The device also has low power and moderate gas consumption (several L/min) and provides surface modification on both sides of planar polymer samples [27]. Moreover, 3D objects (like seeds or small samples) can be placed inside the funnel and treated on all sides simultaneously [26,27]. The beneficial properties of this jet device can be explained by the effective trapping of the reactive species inside the conical horn that can be achieved at very short (few mm) sample-to-horn exit distances and moderate gas flow rates [28].

In this work, we report on the investigation of an atmospheric plasma jet terminating with a 70 mm diameter conical nozzle that is covered by a woven fabric. The latter improves the device’s operation so that it can operate at a wide range of Ar flow rates and gap distances, and produces a stable plasma under the entire area of the funnel exit. Therefore, the proposed plasma jet device is suitable for applications that require uniform surface modification over a relatively large area.

The polymer chosen in this work was polyethylene (PE), a versatile polymer, that finds numerous applications across various industries due to its desirable properties such as high chemical resistance, low electrical conductivity, lightweight nature, and cost-effectiveness [29]. As such a popular polymer, many studies are conducted to develop or improve its processing, including plasma treatments, both at low pressure and atmospheric pressure, using various types of devices such as, for example, DBD discharges and APPJs [29,30,31,32,33,34].

## 2. Materials and Methods

**Figure 1:**
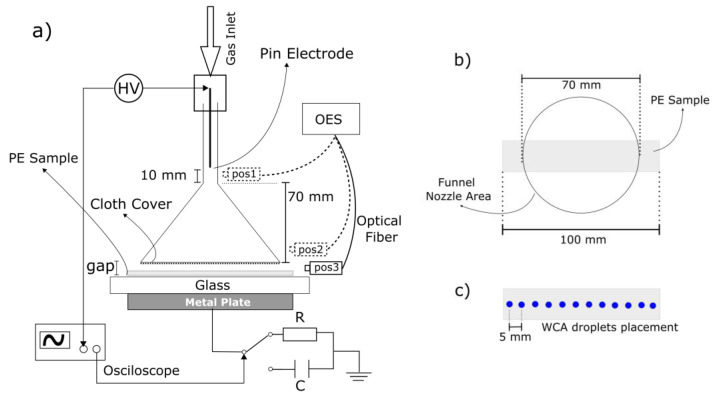
(**a**) Schematic drawing of the plasma jet configuration; (**b**) Top view scheme of the PE sample position during treatment; (**c**) Scheme of droplet distribution for WCA measurements.

A schematic drawing of the plasma jet configuration employed in this work is shown in Figure 1. It consists of a pin electrode (2.4 mm thick tungsten rod) centered inside a 2.5 mm thick quartz conical funnel, which has inner diameters of 4 mm at the straight part and 70 mm and at the funnel exit, respectively. The device was installed vertically, with its wider orifice pointing downward. The bottom of the funnel exit was completely covered by a 0.5 mm thick plain-woven fabric composed of intertwined (90°) cotton fibers, with a density of 150 treads/square inch (from Indústria Têxtil Nossa Senhora do Belém LTDA, Itatiba, Brazil). The cotton fabric allows for gas to pass through it also, and helps improve the gas flow distribution across the funnel exit. Beneath the funnel, a grounded metal electrode (Ø 155 mm) covered by a 3 mm thick glass slab was placed, on top of which the PE samples were placed. The distance from the funnel bottom to the glass slab varied from 0 to 10 mm. Argon gas (99.996% purity from AirLiquide, Cuiabá, Brazil) was admitted into the system through a Teflon holder on the funnel top end. The gas flow rate was adjusted ranging from 0 to 4 SLM by a Horiba model STEC N100 (Horiba, Ltd., Kyoto, Japan) mass-flow controller. Plasma was excited by a commercial AC power supply (Minipuls 6, GBS Elektronik, Dresden, Germany), which was operated in burst mode. The generated voltage waveform was an amplitude-modulated sinewave, i.e., a sequence of N high-voltage oscillations is followed by a voltage off-period. The number of high-voltage cycles (N = 12), the signal frequency (25.0 kHz), and the burst repetition period (Tr = 2.0 ms) were kept constant, while the voltage magnitude and discharge current changed depending on the discharge parameters.

The charge transferred to the target and the discharge current were obtained by measuring the voltage drop across a serial capacitor of 10 nF or a serial resistor of 100 Ω, respectively. The high-voltage signal applied to the pin electrode was measured by a P6015A Tektronix voltage divider (1 × 1000). The signals were monitored on a digital oscilloscope (TDS3032C model from Tektronix, Beaverton, OR, USA). To determine the plasma jet mean power, we used the Q-V Lissajous method, which was adapted for amplitude-modulated voltage signals, as reported in previous work [26].

For all operation conditions, plasma was generated in the form of randomly distributed filaments that commenced from the pin electrode, propagated down the quartz funnel, and after passing through the cloth, reached the glass plate over the grounded electrode. Although permeable, the cloth keeps an Ar-enriched atmosphere inside the conical horn, which facilitates the discharge ignition. Therefore, the cloth cover allows for device operation with bigger gaps between the exit nozzle and the target. For instance, in previous works [26,27], where a similar device was operated without the cloth cover, plasma could not be excited at gaps bigger than 3 mm.

Optical emission spectroscopy was performed in three regions: on the edge of the pin electrode, above the cloth inside the quartz funnel, and right below the cloth (as shown in Figure 1, pos1, pos2, and pos3, respectively), to investigate how the cover interferes with the propagation of the species generated by the plasma. For these measurements, an Avantes (Apeldoorn, Netherlands) spectrometer model AvaSpec-ULS-RS-TEC was used.

It is known from the literature that the treatment of polymers by atmospheric plasmas results in enhanced wettability and adhesion, properties that are very much desirable for coating, printing, painting, and dying [35,36]. All these applications require plasma sources that can generate uniform plasma over a large area. For initial tests, we employed polyethylene (PE) as a common engineering polymer. The plasma treatments were performed on 0.960 g/cm^3^, 1.0 mm thick commercial PE (from LAMIEX INDUSTRIA DE PLASTICOS LTDA., Pinhais, Brazil). The samples were cut in rectangular shapes with a 100 × 20 mm size. The sample was placed below the device’s nozzle, as shown in Figure 1b. It is important to note that the length of the samples is greater than the diameter of the funnel so that measurements could be taken along the sample to check the homogeneity of the treatment and verify whether any changes occur beyond the funnel exit geometric area. To remove organic contaminants, all samples were ultrasonically cleaned first in distilled water for 15 min, and after that in isopropyl alcohol for 10 min and finally left to dry at room temperature overnight.

To assess the surface modification of polymer samples induced by the conical plasma jet, we employed water contact angle (WCA) measurements. They were performed using the sessile drop method on a Rame-Hart goniometer (300 F1) with an automated drop dispenser. Deionized water was used as a test liquid and the volume of each drop was set to 1.0 μL. For each sample, a sequence of equidistant droplets was deposited on the sample surface to determine the treatment area and the radial distribution of the WCA, as shown in Figure 1c. The WCA measurements were performed within an interval of 10 min after the plasma treatment, and both faces of the samples, the top (facing the funnel nozzle) and bottom (facing the glass dielectric), were analyzed; the measurement along each surface took about 3 min to be performed.

To evaluate the functionalization of the sample surface, XPS analysis was performed with Kratos AXIS Supra (Kratos Analytical, Manchester, UK). For these measurements, it was assumed that the treatment is symmetrical, so after being treated, the samples were cut in half (50 × 20 mm) and one half was analyzed at the top face and the other half at the bottom. Longitudinal scans with a 2 mm sampling interval of these samples gave us the values of their elemental composition from the edge to the center.

To evaluate the possible modifications of surface morphology, SEM measurements were performed by a Carl Zeiss (Oberkochen, Baden-Württemberg, Germany) EVO LS 15 at a low pressure (10^−3^ Pa), and electron high tension (EHT) of 5.0 kV. Before the measurements, the samples were covered by a 6 nm thick gold layer deposited via sputtering. Topography images were taken from both the top and bottom faces of the samples, at the center and the border of the treated samples.

## 3. Results

**Figure 2:**
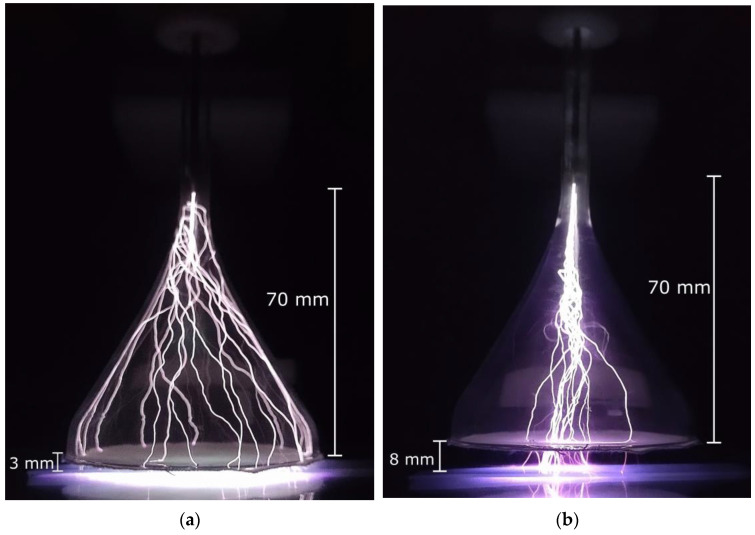
Photos of the funnel device operating with an argon flow of 4.0 SLM and gap distance of (**a**) 3.0 mm, and (**b**) 8.0 mm.

The porous fabric at the funnel exit greatly improves the gas flow dynamics as well as discharge homogeneity, thus allowing for device operation over a wide range of gas flow rates (1–4 SLM) and distances (0–10 mm). Only in extreme cases, like the combinations of the largest gaps with very low Ar flows, the discharge is not stable. The device operating with 4.0 SLM of argon with two different gaps of 3 mm and 8 mm is shown in Figure 2a,b, respectively. It can be observed that by increasing the gap, the discharge filaments tend to stop running along the walls of the funnel and go straight to the glass plate that covers the second electrode.

**Figure 3:**
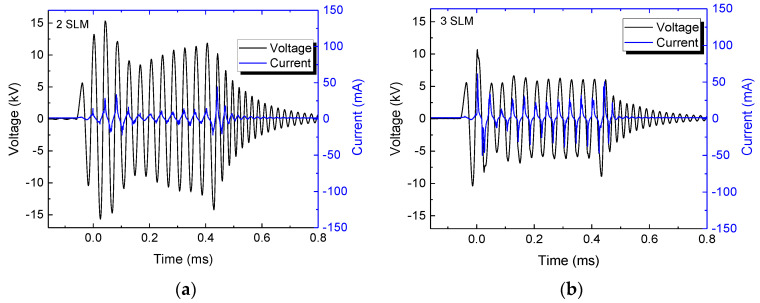

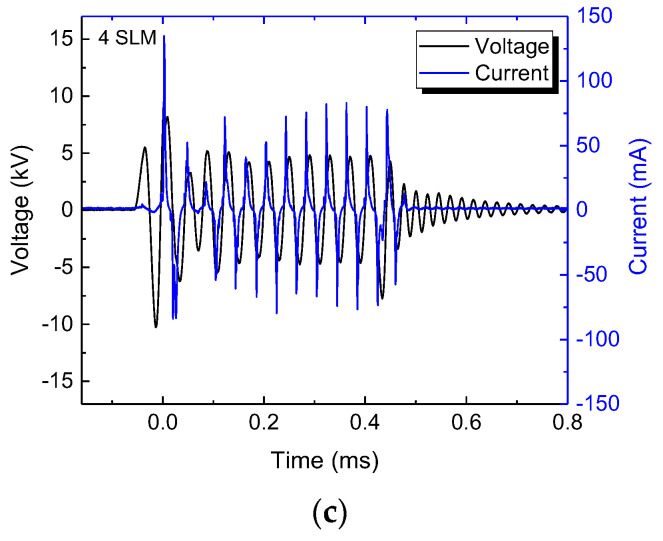
Typical current/voltage waveforms for discharge with a 5.0 mm gap and argon flow of (**a**) 2.0 SLM, (**b**) 3.0 SLM, and (**c**) 4.0 SLM.

**Figure 4:**
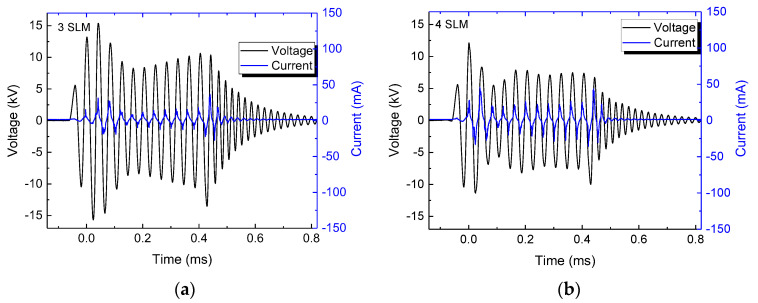
Typical current/voltage waveforms for discharge with an 8.0 mm gap and argon flow of (**a**) 3.0 SLM and (**b**) 4.0 SLM.

The distance to the target, as well as the gas flow rate can affect the plasma jet operation, and more specifically, its current waveform and mean power. The typical current and voltage waveforms at different gas flow rates are shown in Figure 3a–c for a gap distance of 5.0 mm, and in Figure 4a,b for an 8 mm gap. In the latter case, it was not possible to ignite the discharge at a gas flow of 2.0 SLM. It can be seen from them that higher gap values require a higher argon flow to obtain stable voltage/current waveforms, which is evidenced by little variations in the signal amplitude within the burst. Moreover, as can be seen in Figure 3 and Figure 4, the current amplitude increases with the argon flow, while conversely, the voltage magnitude first decreases rapidly and after that, it tends to saturate at higher flows.

**Figure 5:**
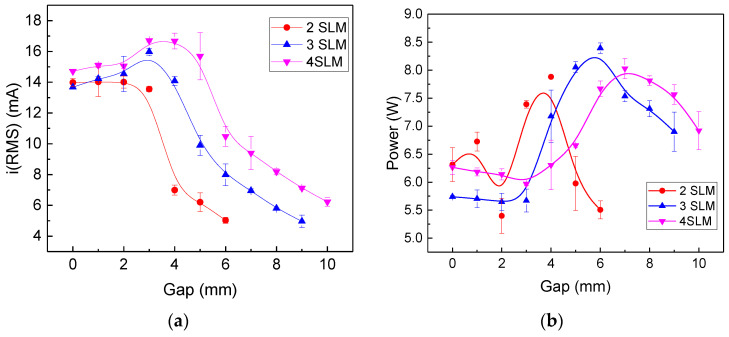
Influence of the gap distance for different Ar flow rates on (**a**) rms currents, and (**b**) discharge power.

Figure 5 depicts the influence of gap distance for different Ar flow rates on (a) the RMS discharge current, and (b) the discharge power. It is possible to observe that for each gas flow rate, there is an optimum gap distance where the power reaches its maximum. Also, for the gas flow rates in Figure 5a, the RMS current is relatively stable up to a certain gap value, beyond which it decreases sharply, and the power maximum occurs right after this fall. This shift in the maximum of the RMS current and voltage signals is probably due to the nonmonotonic behavior of the applied voltage signal at different gaps.

**Figure 6:**
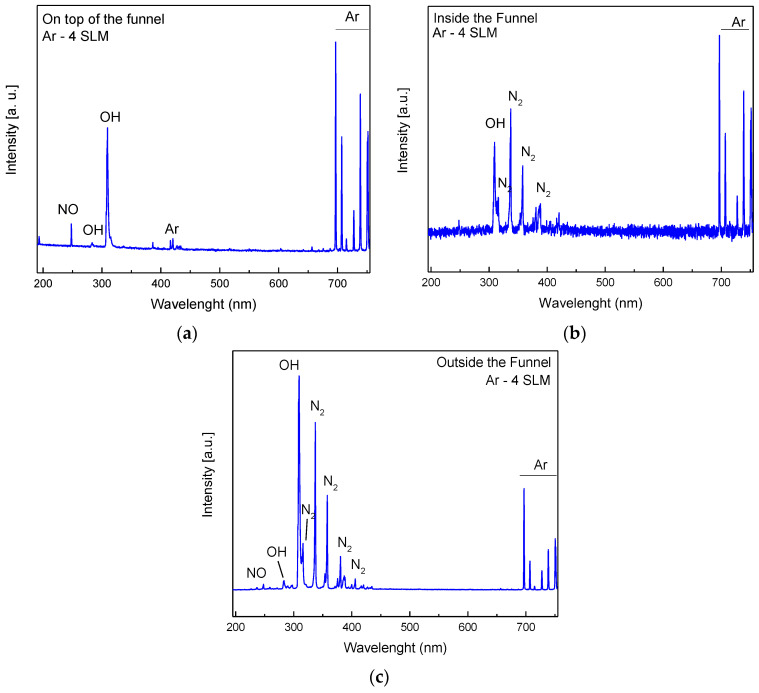
OES for discharges with a 4.0 mm gap (**a**) inside, on top of the funnel with 4.0 SLM of argon flow, (**b**) inside the funnel (right above the cloth) with 4.0 SLM of argon flow, and (**c**) outside the funnel with 4.0 SLM of argon flow.

The results of optical emission spectroscopy for an Ar flow of 4.0 SLM and gap distance of 4 mm are presented in Figure 6. the optical fiber position for each measurement is depicted in Figure 1 indicated as pos1, pos2, and pos3, corresponding, respectively, to Figure 6a–c. To evaluate the effect of permeable cotton fabric on the composition of the generated species, the spectra were acquired in two places inside the funnel, in the funnel narrow part close to the Ar admission (Figure 6a), and right above the cloth in the funnel large part (Figure 6b). Figure 6c shows the OES spectrum collected right below the cloth. As can be seen, the spectra inside the funnel are dominated by the intense argon spectral line, which means that the cloth can hold an argon-enriched atmosphere inside the device. This is an important role that facilitated the discharge ignition and allowed for the device operation with bigger gaps. Alongside the Ar lines, the spectrum in Figure 6a presents a few molecular lines, mostly OH emissions coming from moisture inside the gas line. However, the spectrum taken close to the permeable cloth (Figure 6b) shows the characteristic emission bands of excited N_2_ molecules, which indicate Ar mixing with air. On the other hand, the spectrum under the cloth shown in Figure 6c exhibits some weaker atomic lines coming from Ar expelled through the cloth, and is dominated by the typical molecular emissions coming from excited N_2_, OH, and NO molecules that are commonly generated in atmospheric pressure plasma jets [37].

**Figure 7:**
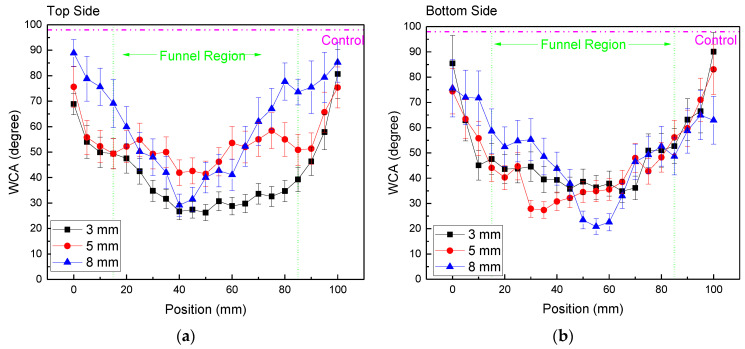
WCA distribution of PE samples treated with an argon flow of 4.0 SLM and different gap values for (**a**) the top side of the sample, and (**b**) the bottom side of the sample.

The PE samples were exposed to plasma for 5.0 min and after that subjected to wettability analysis. A series of WCA measurements were performed with a 5 mm spacing between the consecutive drops to obtain the treatment profile along the target’s long side, as shown in Figure 1c. The top and bottom faces of each sample were measured. As noted in previous work [27], when applying a plasma jet to a thin dielectric sample, a secondary DBD-type discharge is generated between the sample bottom and the sample holder. Figure 7 depicts the water contact angle distributions for different gap values, while keeping an argon flow of 4.0 SLM. The samples were larger than the funnel diameter and stretched beyond the funnel edge. As can be seen in Figure 7, the plasma modification effect happened within the entire funnel geometrical area, as well as in its close vicinity. Also, as in [27], both sides of the samples were treated. Except for the big gap of 8 mm, which is discussed separately, within the funnel region, the bottom and top sides of the samples exhibit similarly wide plateau-like WCA distributions. As the sample’s top face is in direct contact with the Ar plasma jet, the degree of surface modification is higher at a shorter gap distance (d = 3 mm). On the other hand, the bottom side of the sample has approximately the same WCA for gap distances of 3.0 and 5.0 mm. As shown in Figure 2b, for bigger gaps, the discharge filaments tend to concentrate in the middle of the funnel, thus causing more intense treatment in that region. Therefore, the contact angle distribution obtained for the 8.0 mm gap (blue triangles in Figure 7a,b) is not uniform along the sample, exhibiting a triangular shape with a minimum roughly at the funnel center. Another observation is that on the top side of the sample, the wettability remains relatively unchanged for about another 10 mm beyond the funnel geometrical region, while for the bottom side, the WCA distribution starts decreasing more abruptly. This finding can be explained by the fact that reactive species formed on the top side of the sample are carried by the Ar flow outside the funnel, where they can still alter the polymer surface characteristics. On the other hand, the reactive species produced under the sample by the secondary DBD discharge can expand only due to diffusion, thus covering a smaller area.

**Figure 8:**
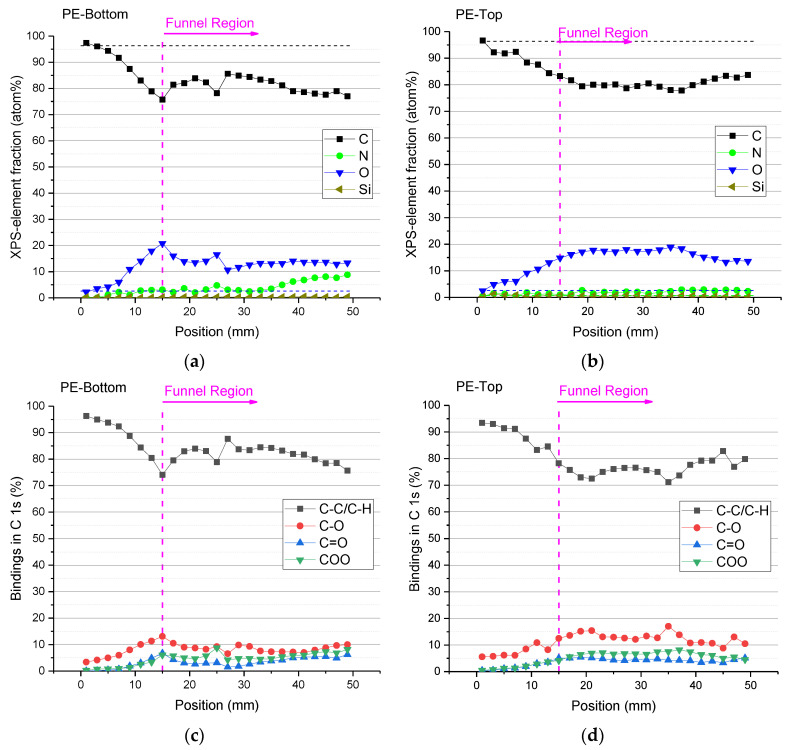
Spatially resolved XPS results of PE samples treated for 5 min with a gap of 5 mm and argon flow of 4.0 SLM. Element fractions on the (**a**) bottom side and (**b**) top side of the sample. Carbon bindings on (**c**) the bottom side and (**d**) top side of the sample.

**Figure 9:**
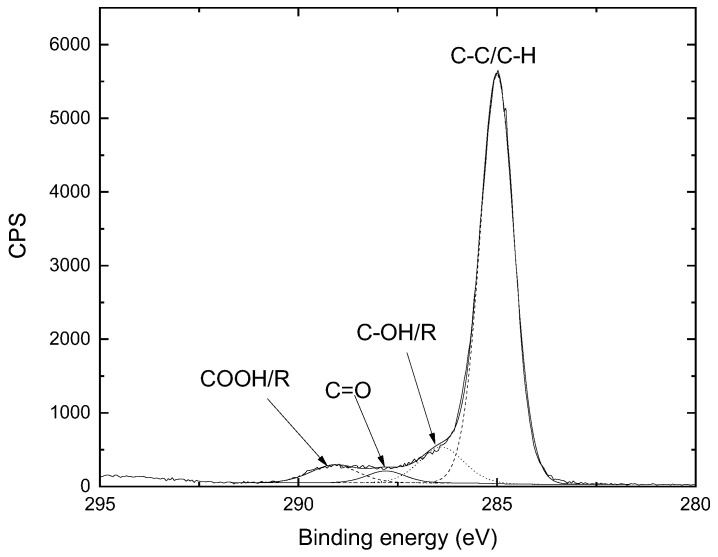
Example of an XPS spectra deconvolution, taken in the position of 19 mm on the top side. C 1s peaks were fitted using four components: C–C/C–H @ BE 285.0 eV (calibr.), C–OH/R @ BE 286.5 ± 0.2 eV, C=O @ BE 287.8 eV, and COOH/R @ BE 289.2 ± 0.2 eV.

The results from XPS analysis of PE samples treated for 5 min using a 5.0 mm gap and argon flow of 4.0 SLM are presented in Figure 8. The treated samples were cut in half and the left-hand part of the sample was analyzed on its top face, while the right-hand part was measured on its bottom face. The spatially resolved XPS measurements were taken along the sample’s central line with a 2.0 mm separation, starting from the edge of the sample to its center, which coincides with the center of the funnel. The elemental composition of the untreated PE sample is 96 at% C and 4 at% O due to surface oxidation. In Figure 9, an example of an XPS C 1s spectrum is given. This measurement was taken from position 19 mm on the top side, and the C 1s peaks were fitted using four components: C–C/C–H @ BE 285.0 eV (calibr.), C–OH/R @ BE 286.5 ± 0.2 eV, C=O @ BE 287.8 eV, and COOH/R @ BE 289.2 ± 0.2 eV [38,39,40]. Figure 8a,b shows the distribution of the elemental composition of both faces of the treated PE sample. It is possible to see a reduction in the carbon content and the appearance of oxygen on both sides of the treated sample. Traces of Si (due to sample contamination) and a small amount of N (<3 at%) were also detected. However, a significant percentage of nitrogen atoms (up to 8 at%) was found only on the bottom side of the sample (Figure 8a). This is probably due to the nature of the secondary air DBD discharge formed between the sample and the sample holder. As shown in the literature [32], plasma treatments of polymers in the air usually result in the incorporation of oxygen and nitrogen atoms into the material surface [41].

In Figure 8c,d, the binding in the C 1s peaks on both sample faces is represented. It is possible to observe that as one approaches the funnel region, there is a gradual reduction in the C–C and C–H bonds and the emergence of oxygenated groups such as C–O, C=O, and COO on the surface. When entering into the funnel region, the amount of these functional groups tends to remain practically unchanged throughout the sample. It is worth mentioning that the reduction in functional groups, as well as the O/C atom contents outside the funnel region is more abrupt on the bottom side of the sample than the ones on the top samples side. This finding also corroborates with the WCA distributions.

**Figure 10:**
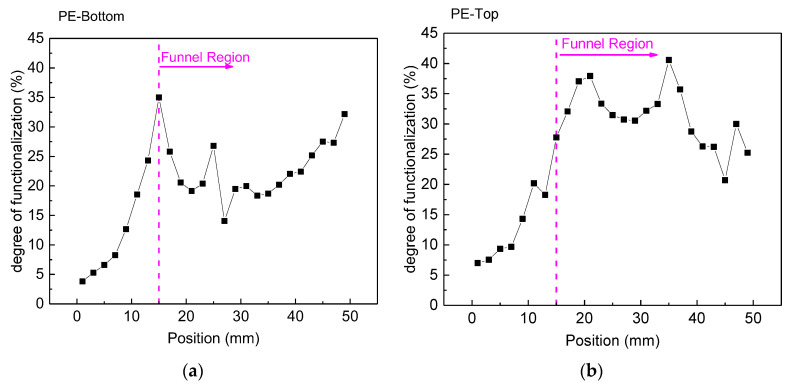
Degree of surface functionalization of the PE samples obtained via XPS (**a**) on the bottom side of the sample and (**b**) the top side. The samples were treated for 5 min with a gap of 5.0 mm and an argon flow of 4.0 SLM.

The plasma treatment can modify the chemistry of polymeric surfaces by adding functional groups [42]. This functionalization is observed mainly with the addition of oxygen and nitrogen groups. The extent of PE surface functionalization was determined using XPS, and it is depicted in Figure 10, where the degree of functionalization is shown. The degree of functionalization provides information about the ratio of the sum of functional groups (-OH/R, =O, -COOH/R) to C–C/C–H [38,39,40]. Again, it is possible to see in Figure 10a that at the end of the funnel region, there is a sharp decay in the degree of functionalization for the bottom of the sample. On the upper face of the sample, the corresponding functionalization decay is smoother (Figure 10b). Moreover, within the funnel area, the top PE side exhibits higher functionalization levels of approximately 35%, whereas the corresponding values for the bottom sample face are around 25%. This is in good agreement with the WCA measurements that exhibited similar trends. It should be noted, however, that the functionalization of the upper sample side is predominantly attributed to oxygen groups, whereas in addition to oxygen groups, the lower side also exhibits the presence of nitrogen functional groups.

**Figure 11:**
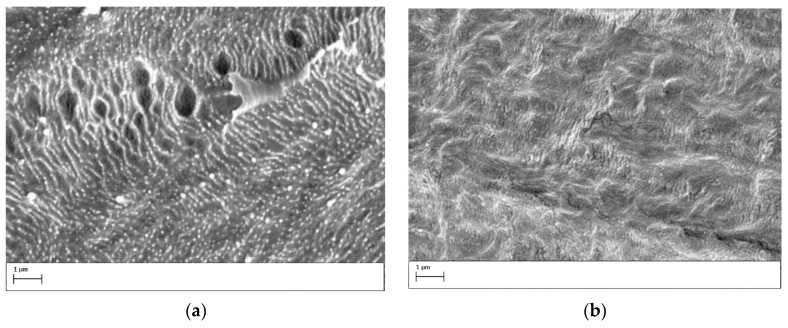

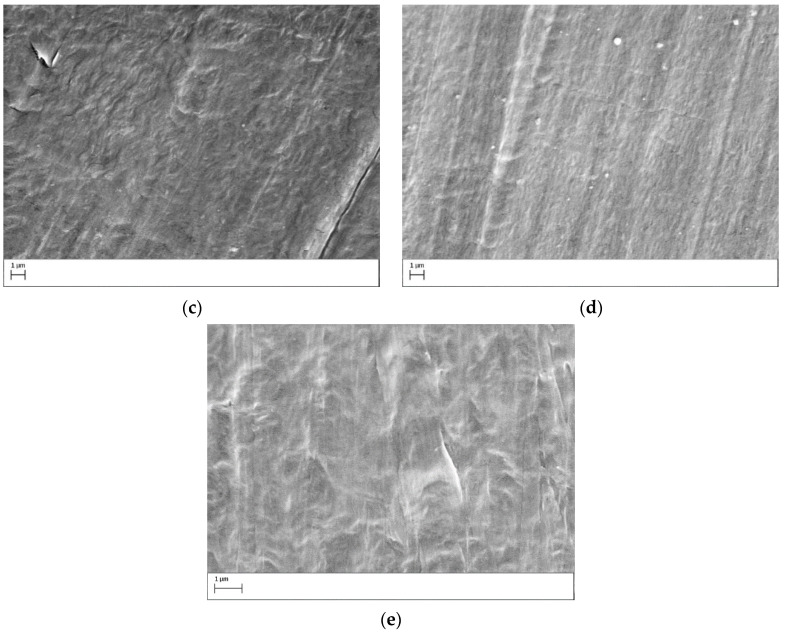
SEM images of the PE samples treated for 5 min with a gap of 5.0 mm and an argon flow of 4.0 SLM. The analysis was performed at (**a**) the central region on the bottom side of the sample, (**b**) the central region on the top side of the sample, (**c**) the border on the bottom side of the sample, (**d**) the border on the top side of the sample, and (**e**) the center of the untreated sample.

The results from the SEM analyses are presented in Figure 11. It shows typical micrographs of the bottom and top side of the PE sample taken in the central region (Figure 10a,b), respectively, and the edge region (Figure 11c,d) after a 5 min plasma treatment with an argon flow of 5.0 SLM and an 8.0 mm gap. For comparison, Figure 11e depicts the surface of the untreated PE. First, in the edge region on both sides of the sample (Figure 11c,d), no changes in the surface morphology can be observed when compared to the untreated sample (Figure 11e). This observation agrees with the finding that at large gaps, the discharge is mostly concentrated at the funnel central part, and consequently, less intense surface modification happens at the funnel edge. On the other hand, in the central region of the treated sample (Figure 11a,b), some significant changes can be seen. On the top side of the treated sample (Figure 11b), only mild morphological alterations, probably due to the etching and removal of surface contaminants, can be seen. However, Figure 11a clearly shows the formation of small round structures in the central part on the bottom face of the PE sample. These structures consist of loosely bonded short polymer fragments that are known as low molecular-weight oxidized materials (LMWOMs). Their formation is typically observed on the surface of polymers treated in air plasma [41,43,44], thus corroborating the existence of the secondary DBD discharge formed under the PE sample.

## 4. Conclusions

The funnel-shaped plasma jet has demonstrated potential for uniform treatment over large areas when compared to conventional plasma jets, without the requirement of high gas flow rates. By employing a cloth cover on the exit nozzle, the device has been able to operate at increased distances from the sample holder, thereby expanding its potential applications in material treatments. The device was characterized by electrical and optical measurements, showing that device power consumption and proportions between the excited reactive species can be optimized through the careful choosing of the operation parameters, such as gas flow rate and gap distance. It has been observed that the device operation is influenced by variations in the flow rate and gap, with greater distances leading to a reduction in the uniform part of the treated area.

Simultaneous treatment of both sides of the sample has been successfully achieved, which offers a time-saving advantage in situations where the treatment of both surfaces is necessary. Specifically, the upper face is subjected to direct treatment from the discharge emanating from the funnel, while the lower face is treated by a secondary DBD discharge. However, despite the simultaneous treatment, differences exist between the surface modification effect on the two sides of the PE sample. The bottom side presented the incorporation of nitrogen groups and the formation of LMWOM, while the upper sample face exhibited a more intense incorporation of O-containing groups. Furthermore, it is worth noting that the treatment of the upper side of the samples occurs over a broader area than that covered by the funnel, while the treatment of the lower side is confined to a few millimeters beyond this region. These observations contribute to a deeper understanding of the operational characteristics and limitations of the funnel-shaped plasma jet device, facilitating its implementation in various material treatment scenarios.
